# Exploration of the Causal Association Between Behavioral Risk Factors and Gallstone Disease Development in Two European Ancestry Populations

**DOI:** 10.7759/cureus.37110

**Published:** 2023-04-04

**Authors:** Khalid O Alyahyawi, Mohammad A Jareebi, Othman A Iskander, Jamaludeen A Othman, Abdulaziz A Alagsam, Waseem S Borik, Mohammed Y Qaarie, Ibrahim M Gosadi

**Affiliations:** 1 Department of Surgery, Faculty of Medicine, Jazan University, Jazan, SAU; 2 Department of Family and Community Medicine, Faculty of Medicine, Jazan University, Jazan, SAU; 3 Department of Anesthesia and Critical Care, Jazan University Hospital, Jazan, SAU; 4 Ophthalmology Center, Prince Mohammed bin Nasser Hospital, Jazan, SAU

**Keywords:** behaviour, mendelian randomization, genetic, risk, gallstone

## Abstract

Introduction

Risk factors for developing gallstones are related to disturbances in either cholesterol or bilirubin metabolism in the biliary tract. The risk of forming gallstones can be associated with chronic illnesses, dietary habits, reduced gallbladder motility, and medications. Our study aims to explore the causal relationship between multiple risk factors, including nutritional habits (cheese intake, salad intake, processed meat intake, coffee drinking), smoking behavior, overall obesity measured by body mass index (BMI), lipid biomarkers, total bilirubin and maternal diabetes mellitus (DM) and the development of gallstone disease in two different populations of European ancestry (United Kingdom Biobank (UKB) and FinnGen).

Materials and methods

Using publicly available genome-wide association studies (GWAS) data, we performed a two-sample Mendelian randomization (MR) to examine the association between risk factors and gallstone development. Exposures used in this study included age of smoking initiation, smoking intensity, coffee intake, cheese intake, salad intake, processed meat intake, BMI, and lipid biomarkers (cholesterol, low-density lipoproteins (LDL), triglycerides (TG), and high-density lipoproteins (HDL)). Current analyses were based on 93 single nucleotide polymorphisms (SNPs) for smoking initiation, four SNPs for smoking intensity, 65 SNPs for cheese intake, three SNPs for coffee intake, 22 SNPs for salad intake, 23 SNPs for processed meat intake, 79 SNPs for BMI, 26 SNPs for maternal DM, 89 SNPs for total bilirubin, 46 SNPs for cholesterol, 41 SNPs for LDL, 55 SNPs for TG, and 89 SNPs for HDL. The outcome in this study is gallstones/cholelithiasis. To evaluate the causal relationships between these risk factors and gallstones, two-sample MR methods were used. *TwoSampleMR* package in R software version 4.0.5 (R Foundation for Statistical Computing, Vienna, Austria) was used to obtain MR analyses and sensitivity analyses.

Results

In the UKB, genetic predispositions to smoking initiation, BMI, and total bilirubin were significantly associated with an increased risk of gallstones. The odds of gallstones would increase per 1-SD increase of genetically estimated smoking initiation (OR: 1.004, P=0.008), BMI (OR: 1.02, P<0.001), and total bilirubin (OR: 1.0001, P=0.025). Conversely, genetic predispositions to cheese intake, coffee intake, cholesterol, LDL, and TG were statistically significantly associated with a decreased risk of gallstones (OR=0.99, P=0.014; OR=0.97, P=0.009; OR=0.99, P=0.006; OR=0.99, P=0.01; and OR=0.99, P<0.001, respectively).

In FinnGen, genetic predispositions to BMI and total bilirubin were significantly associated with an increased risk of gallstones. The odds of gallstones would increase per 1-SD increase of genetically estimated BMI (OR: 1.7, P<0.001) and total bilirubin (OR: 1.02, P=0.002). Conversely, genetic predispositions to cheese intake, coffee intake, cholesterol, LDL, and TG were statistically significantly associated with a decreased risk of gallstones (OR=0.23, P=0.006; OR=0.42, P=0.041; OR=0.77, P=0.034; OR=0.88, P=0.008; and OR=0.70, P=0.005, respectively).

Conclusion

Genetically estimated BMI and total bilirubin levels were associated with increased risk of gallstones among the two populations while genetically estimated cheese intake, coffee intake, and cholesterol, LDL, and TG levels factors were consistently associated with reduced risk of gallstones among the two populations.

## Introduction

Gallstone is a stone formed from the precipitation of bile components. They can develop inside the gallbladder or in the bile tract. Gallstones are mainly classified into two types: cholesterol stones and bilirubin pigment stones. Cholesterol gallstones are the most common type comprising approximately 90% of them [1]. Nonetheless, there are other less common forms of stone, including black pigment stones which are usually caused by chronic hemolysis, and brown pigment stones which are usually formed in the infected bile ducts.

Studies that utilized ultrasound to assess the presence of gallstones indicated a mean prevalence rate of 10-15% in adult European, and of 3-5% in African and Asian populations [2]. Variation in the prevalence of gallstones between populations suggests the presence of a genetic and ethnic variation effect influencing the variation of the epidemiology of gallstone disease from a global perspective.

The clinical presentation of gallstones can vary from being asymptomatic to having serious complications. It has been reported that the proportion of gallstone carriers who are not aware of their clinical condition can reach 80% of gallstone carriers [3, 4]. Only symptomatic gallstones are considered gallstone disease, which can be associated with various clinical presentations and complications. Every year about 1-2% of patients diagnosed with gallstone disease develop complications and require surgery [5]. Common complications that can occur after a cholecystectomy include cholangitis and pancreatitis as well as gallstone recurrence. The frequent recurrence of gallstones can affect the quality of life and the health status of the patients [6].

Risk factors for the development of gallstones are related to disturbances in either cholesterol or bilirubin metabolism in the biliary tract. The risk of formation of gallstones has been reported to be associated with chronic illnesses, dietary habits, reduced gallbladder motility, and medications. The well-defined risk factors are advanced age (although gallstone-related hospitalizations in the young (< 20 years of age) have increased over time [7]), female sex, and tobacco smoking, which have been shown in multiple observational studies to increase the risk of developing gallbladder disease [8]. Gallstones disease in younger people (< 50 years old) has been suggested to be associated with metabolic syndrome and obesity [9, 10]. Findings based on observational associations between gallstones and other risk factors can be confounded, hence causal inference cannot be established in such associations. For example, diabetes mellitus (DM), obesity, and reduced physical activity are closely interrelated and their exact effects on the development of cholelithiasis and gallstone disease are not known. Therefore, other approaches such as Mendelian randomization can be leveraged to establish the causality between these risk factors and gallstones.

Mendelian randomization (MR) studies allow for the prediction of a causal effect of an exposure on health outcomes. It takes into account the genetic variants that are randomly distributed during genetic development that are potentially involved in the occurrence of an individual's health condition [11]. Our study aims to introduce and explore the causal relationship between multiple risk factors, including nutritional habits (cheese intake, salad intake, processed meat intake, coffee drinking), smoking behavior, overall obesity measured by body mass index (BMI), lipid biomarkers, total bilirubin and maternal diabetes and the development of gallstone disease in two different populations of European ancestry (UK Biobank and FinnGen). Using publicly available genome-wide association studies (GWAS) data, a two-sample MR approach was performed to examine the association between the risk factors and gallstone development.

## Materials and methods

Exposure data

Exposures used in this study included age of smoking initiation, smoking intensity, coffee intake (amount of coffee each day), cheese intake, amount of salad intake, amount of processed meat intake, BMI, and lipid biomarkers (cholesterol, low-density lipoproteins (LDL), triglycerides (TG), and high-density lipoproteins (HDL)) [12]. These measures were chosen based on the publicly available data of the genome-wide significant single nucleotide polymorphisms (SNPs) from different consortia. These consortia included the UK Biobank (UKB), GWAS and Sequencing Consortium of Alcohol and Nicotine use (GSCAN), Genetic Investigation of ANthropometric Traits (GIANT), and Global Lipids Genetics Consortium (GLGC) [13]. The SNPs are the genetic variants in the human DNA that have been found to predict certain traits [14]. These associations were based on GWAS level significance (P<5x10-8). Current analyses are based on 93 SNPs for smoking initiation, four SNPs for smoking intensity, 65 SNPs for cheese intake, three SNPs for coffee intake, 22 SNPs for salad intake, 23 SNPs for processed meat intake, 79 SNPs for BMI, 26 SNPs for maternal DM, 89 SNPs for total bilirubin, 46 SNPs for cholesterol, 41 SNPs for LDL, 55 SNPs for TG, and 89 SNPs for HDL.

Outcome data

The outcome in this study is gallstones/cholelithiasis. This variable is a binary outcome of either having the condition or not. The genetic data (SNPs) for gallstones were obtained from two populations: UKB and FinnGen (Finnish health research environment for genomic research) [15,16]. The SNPs for gallstones per each exposure (after harmonization) were as follows (for both populations): 92 SNPs for smoking initiation, four SNPs for smoking intensity, 64 SNPs for cheese intake, three SNPs for coffee intake, 22 SNPs for salad intake, 23 SNPs for processed meat intake, 79 SNPs for BMI, 84 SNPs for total bilirubin, 46 SNPs for cholesterol, 41 SNPs for LDL, 55 SNPs for TG, and 89 SNPs for HDL.

Statistical analysis

To evaluate the causal relationships between the risk factors and gallstones, two-sample MR methods were used. *TwoSampleMR *package in R software version 4.0.5, (R Foundation for Statistical Computing, Vienna, Austria)) was used to obtain MR analyses and sensitivity analyses. The steps of the analysis began with the extraction of genetic data for exposures, followed by the retrieval of outcome data based on the exposure data, then data harmonization was performed to ensure the quality of matched alleles in two independent datasets, and finally performing the MR analysis [17]. In MR analyses, a P-value of less than 0.05 was regarded as statistically significant. All MR findings were based on Inverse variance weighted (IVW). However, all MR measures, including Mendelian randomization-Egger (MR-Egger), were also inspected for any major deviation from IVW findings.

## Results

The number of SNPs varied between four and 93 with a total of 661 SNPs for all risk factors. These SNPs were obtained from different consortia with sample sizes ranging from 23205 to 607291 per risk factor (Table 1). These studies investigate the relationship between specific genetic variants and various health-related traits. The Genetics of Smoking Consortium (GSCAN) investigated genetic variants related to smoking initiation, involving 93 SNPs and a sample size of 607,291 individuals. The study aimed to identify genetic factors that contribute to the age at which individuals start smoking. Another study by the Within-family GWAS consortium looked at the level of smoking intensity and its association with genetic variants. This study involved four SNPs and a sample size of 23,205 individuals.

**Table 1:** Summary of risk factors BMI: body mass index; DM: diabetes mellitus ; GIANT: The Genetic Investigation of ANthropometric Traits; GLGC: The Global Lipids Genetics Consortium; GSCAN: GWAS & Sequencing Consortium of Alcohol and Nicotine Use; GWAS: Genome-Wide Association Studies; HDL: high-density lipoproteins; LDL: low-density lipoproteins; SNPs: single nucleotide polymorphisms; TG: triglycerides; UKB: UK Biobank

Exposure	No. of SNPs	Sample size	Population (consortium)
Smoking initiation	93	607291	GSCAN
Smoking intensity	4	23205	Within family GWAS consortium
Cheese intake	65	451486	UKB
Salad intake	22	435435	UKB
Processed meat intake	23	461981	UKB
Coffee intake	3	64949	UKB
BMI	79	339224	GIANT
Maternal DM	26	423892	UKB
Total bilirubin	89	342829	UKB
Cholesterol	46	92260	GLGC
LDL	41	83193	GLGC
TG	55	177861	GLGC
HDL	89	187167	GLGC

The UK Biobank (UKB) conducted three different studies investigating the impact of genetic variants on dietary habits. The first study involved the consumption of cheese and looked at 65 SNPs and a sample size of 451,486 individuals. The second study explored genetic variants associated with the consumption of salad, involving 22 SNPs and a sample size of 435,435 individuals. The third study investigated genetic variants related to the consumption of processed meat and coffee. It involved 23 SNPs and three SNPs, respectively, and a sample size of 461,981 individuals for processed meat and 64,949 individuals for coffee.

The Genetic Investigation of ANthropometric Traits (GIANT) consortium studied genetic variants related to body mass index (BMI), with a sample size of 339,224 individuals and 79 SNPs. The study aimed to identify genetic factors that may contribute to differences in BMI between individuals. Another UKB study explored genetic variants related to maternal diabetes, involving 26 SNPs and a sample size of 423,892 individuals.

Finally, the Global Lipids Genetics Consortium (GLGC) investigated the relationship between genetic variants and lipid-related traits. The first study looked at genetic variants associated with cholesterol levels and involved 46 SNPs and a sample size of 92,260 individuals. The second study explored genetic variants related to low-density lipoprotein (LDL) cholesterol levels, involving 41 SNPs and a sample size of 83,193 individuals. The third study investigated genetic variants associated with triglyceride levels and involved 55 SNPs and a sample size of 177,861 individuals. The fourth and final study explored genetic variants related to high-density lipoprotein (HDL) cholesterol levels and involved 89 SNPs and a sample size of 187,167 individuals.

The gallstones variable was obtained from two independent populations: UKB and FinnGen. An overview of the gallstones variable in each population is shown in Table 2.

**Table 2:** Characteristics of gallstones variable UKB: UK biobank; FinnGen: Finnish health research environment for genomic research

Population	Sample size	Cases	Control
UKB	462933	7682	455251
FinnGen	215027	19883	195144

Findings of gallstones in the UKB consortium

In the UKB consortium, the genetic predispositions to smoking initiation, BMI, and total bilirubin variables were statistically significantly associated with increased risk of gallstones. The odds of gallstones would increase per 1-SD increase of genetically estimated smoking initiation (OR: 1.004, P=0.008), BMI (OR: 1.02, P<0.001), and total bilirubin (OR: 1.0001, P=0.025). Conversely, the genetic predispositions to cheese intake, coffee intake, cholesterol, LDL, and TG were statistically significantly associated with decreased risk of gallstones (OR=0.99, P=0.014; OR=0.97, P=0.009; OR=0.99, P=0.006; OR=0.99, P=0.01; and OR=0.99, P<0.001, respectively). The rest of the associations are summarized in Table 3.

**Table 3:** Summary of two-sample MR results (UKB) BMI: body mass index; DM: diabetes mellitus ; HDL: high-density lipoproteins; LDL: low-density lipoproteins; OR: odds ratio; TG: triglycerides; UKB: UK Biobank; MR: Mendelian randomization

Risk factor	OR	P value
Smoking initiation	1.004	0.008
Smoking intensity	1.001	0.818
Cheese intake	0.99	0.014
Salad intake	0.98	0.117
Processed meat intake	0.99	0.41
Coffee intake	0.97	0.009
BMI	1.02	<0.001
Maternal DM	1.02	0.09
Total bilirubin	1.0001	0.025
Cholesterol	0.99	0.006
LDL	0.99	0.01
TG	0.99	<0.001
HDL	1.001	0.607

Findings of gallstones in the FinnGen consortium

In the FinnGen consortium, genetic predispositions to BMI and total bilirubin variables were statistically significantly associated with increased risk of gallstones. The odds of gallstones would increase per 1-SD increase of genetically estimated BMI (OR: 1.7, P<0.001) and total bilirubin (OR: 1.02, P=0.002). Conversely, genetic predispositions to cheese intake, coffee intake, cholesterol, LDL, and TG were statistically significantly associated with decreased risk of gallstones (OR=0.23, P=0.006; OR=0.42, P=0.041; OR=0.77, P=0.034; OR=0.88, P=0.008; and OR=0.70, P=0.005, respectively). The rest of the associations are summarized in Table 4.

**Table 4:** Summary of two-sample MR results (FinnGen) BMI: body mass index; DM: diabetes mellitus; FinnGen: Finnish health research environment for genomic research; HDL: high-density lipoproteins; LDL: low-density lipoproteins; OR: odds ratio; TG: triglycerides

Risk factor	OR	P value
Smoking initiation	1.04	0.486
Smoking intensity	1.01	0.614
Cheese intake	0.23	0.006
Salad intake	1.06	0.883
Processed meat intake	0.81	0.360
Coffee intake	0.42	0.041
BMI	1.7	<0.001
Maternal DM	1.9	0.151
Total bilirubin	1.02	0.002
Cholesterol	0.766	0.034
LDL	0.88	0.008
TG	0.70	0.005
HDL	0.93	0.347

Gallstones risk between UKB and FinnGen

**Figure 1:**
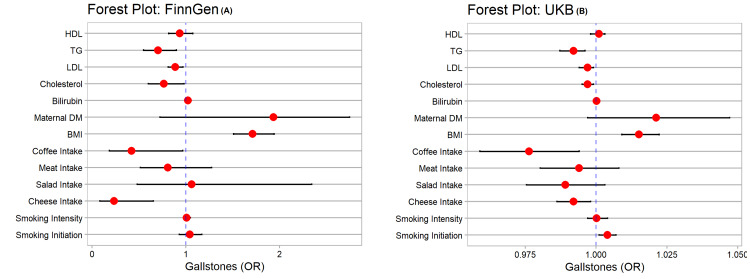
Forest plots of factors associated with gallstones "A": FinnGen findings; "B": UKB findings; BMI: body mass index; FinnGen: Finnish health research environment for genomic research; HDL: high-density lipoproteins; LDL: low-density lipoproteins; OR: odds ratio; TG: triglycerides; UKB: UK biobank

The findings in the two populations were almost the same. However, the risk of gallstones was more prominent among all risk factors in the FinnGen consortium compared to UKB (Figure 1A-1B).

## Discussion

The current investigation utilized the Mendelian randomization method to predict the potential causal effect of exposure to multiple risk factors on the risk of the development of gallstones. Via utilizing GWAS data of the UK Biobank and FinnGen, the association between genetic predisposing of certain clinical characteristics and consumption of certain food items and risk of gallstone was performed. Variations in the risk of development of gallstones according to the estimated genetic risk among the two populations were detected. However, factors that were consistently associated with increased risk of gallstones among the two populations were related to genetically estimated BMI and total bilirubin levels. Furthermore, factors that were consistently associated with reduced risk of gallstones among the two populations were related to the genetically estimated cheese intake, coffee intake, and cholesterol, LDL, and TG levels.

The findings of the current investigation can be compared to similar studies which utilized the Mendelian randomization method to predict factors that can have a causal effect on the development of gallstones. A similar study assessed the risk of gallstone disease among a sample of the UK Biobank (including 10520 cases of gallstone disease), and a sample from the FinnGen consortium (including a sample of 11675 gallstone disease) according to the estimated genetic risk of type 2 diabetes, obesity, and specific lifestyle factors (cigarette smoking, and alcohol and coffee consumption). The study identified an increased risk of gallstone disease with BMI, waist circumference, diabetes, and smoking initiation, and a protective effect of coffee consumption against gallstone disease development [18]. Our investigation also identified a similar association between elevated BMI levels and smoking initiation and the increased risk of gallstone disease, and a protective effect of coffee intake against the development of gallstone disease.

Our investigation identified a causal link between the genetically estimated risk of obesity and the risk of the development of gallstone disease. It is possible to argue that reduced genetically estimated risk of higher obesity levels might provide a protective effect against the development of gallstone disease. Though not measured in our current investigation, the reversibility effect of factors associated with reducing obesity risk on the development of gallstone disease has been indicated by a similar Mendelian randomization study based on the UK Biobank (including a sample of 7682 cases of gallstone disease) and the FinnGen consortium (including a sample of 23089 cases of gallstone disease) which detected a protective effect of the genetically predicted physical activity level against the development of gallstone disease [19]. A potential causal pathway can be postulated where the genetically determined risk of higher physical activity might incur lower obesity risk and thus reduce the risk of gallstones. This suggests the importance of recognizing physical activity and its role in reducing the risk of development of obesity and the subsequent risk reduction of gallstone development.

Several studies have investigated the potential causal pathway between physical activity, obesity, and the risk of developing gallstones. For instance, a large prospective cohort study found that increased physical activity was associated with a reduced risk of developing gallstones, independent of BMI and other potential confounding factors [20]. Another study investigating the genetic determinants of physical activity found that individuals with a higher genetic propensity for physical activity had a lower risk of developing obesity and related metabolic disorders, including gallstones [21]. This study supports the hypothesis that physical activity may reduce the risk of gallstone development by reducing the risk of obesity.

Further evidence for this hypothesis comes from a recent meta-analysis of randomized controlled trials, which found that physical activity interventions led to significant reductions in body weight, BMI, and waist circumference, as well as improvements in metabolic health parameters such as glucose and lipid levels [22]. These findings suggest that physical activity may play a crucial role in reducing the risk of gallstone development through its effects on obesity and related metabolic disorders. Therefore, recognizing physical activity as an important lifestyle factor and promoting regular physical activity may be an effective strategy for reducing the risk of gallstone disease.

Our study identified a protective effect of the genetically estimated coffee intake against the development of gallstone disease. This finding is supported by a similar Mendelian randomization study which utilized the genome-wide association data of European ancestry where a linear relationship was detected suggesting a protective effect of tea and coffee intake against the risk of gallstone disease development but a higher risk of gallstone disease development according to the decaffeinated coffee intake [23]. Another investigation that assessed the risk of gallstone disease according to two genetic variants associated with caffeine consumption indicated a reduced risk of gallstone disease development among subjects with higher coffee consumption propensity [24]. The identified protective association between coffee and tea consumption against the risk of development of gallstone disease but not the consumption of decaffeinated coffee intake might indicate a protective effect of caffeine consumption on the risk of development of gallstone disease and suggests an area for further investigation to identify causal pathways concerning caffeine metabolism and gallstone development.

Our investigation identified a protective effect of the genetically determined lipid profile parameters and risk of development of gallstone disease. Our findings are contradicted by the findings of a similar Mendelian randomization study that assessed the development of gallstone disease according to the genetically predicted LDL, HDL, and TG utilizing the genome-wide association data from the UK Biobank and revealing no evidence of a causal relationship between the genetically predicted lipid profile and gallstone disease [21]. Similarly, another study assessed the risk of gallstone disease according to the genetically determined LDL levels via applying the Mendelian randomization method utilizing eight genetic variants associated with LDL among a Danish population (including 3323 subjects with gallstone disease) and revealed no causal association [25]. It is possible to argue that variation in the selection of variants associated with lipid profile parameters might affect the overall causal effect on gallstone disease development where a larger number of variants were utilized in our investigation in comparison to other similar studies.

Our investigation identified a relatively larger protective effect of genetically predicted cheese intake and risk of development of gallstone disease. This can be considered a novel finding as no similar Mendelian randomization study was detected to indicate a causal association between cheese intake and the risk of gallstone disease. Though there is limited evidence concerning the association between cheese consumption and gallstone development, an association between cheese consumption and the risk of gallstone disease has been indicated in an epidemiological observational investigation. Nonetheless, similar further Mendelian randomization investigations among other populations might be required to strengthen the evidence concerning the protective effect of cheese intake on the risk of developing gallstone disease [25]. 

The current investigation has some limitations that should be taken into consideration. Firstly, the study was based on two population datasets of UK Biobank and FinnGen, which might not be representative of other populations. Secondly, the study only considered genetic predisposition to certain clinical characteristics and consumption of certain food items and did not consider other potential risk factors such as physical activity, stress, and medication use. Thirdly, the study relied on self-reported data for food intake, which could be subject to recall bias. Fourthly, although Mendelian randomization is a powerful method to detect causal relationships, it is still subject to some assumptions and limitations, such as the potential for the pleiotropic effects of genetic variants. Finally, the study did not investigate the potential interactions between the identified risk factors and their joint effect on the risk of gallstone development. Despite these limitations, the study provides valuable insights into the potential causal effects of various risk factors on the risk of gallstone development and highlights areas for further investigation.

## Conclusions

The current study utilized a Mendelian randomization approach and detected a causal effect of the genetically estimated BMI and total bilirubin levels in terms of a higher risk of development of gallstone disease. Conversely, factors that were consistently associated with a reduced risk of gallstones were related to the genetically estimated cheese intake, coffee intake, and cholesterol, LDL, and TG levels. Further investigation is required to elucidate the protective effect of the genetically estimated cheese intake on gallstone development.
